# Assessment of Spillover of Antimicrobial Resistance to Untreated Children 7–12 Years Old After Mass Drug Administration of Azithromycin for Child Survival in Niger: A Secondary Analysis of the MORDOR Cluster-Randomized Trial

**DOI:** 10.1093/cid/ciae267

**Published:** 2024-05-13

**Authors:** Brittany Peterson, Ahmed M Arzika, Abdou Amza, Ramatou Maliki, Alio Mankara Karamba, Mariama Moussa, Mariama Kemago, Zijun Liu, Eric Houpt, Jie Liu, Suporn Pholwat, Thuy Doan, Travis C Porco, Jeremy D Keenan, Thomas M Lietman, Kieran S O’Brien

**Affiliations:** Francis I. Proctor Foundation, University of California, San Francisco, California, USA; Department of Epidemiology and Biostatistics, University of California, San Francisco, California, USA; Centre de Recherche et Interventions en Santé Publique, Birni N’Gaoure, Niger; Programme Nationale de Santé Oculaire, Niamey, Niger; Centre de Recherche et Interventions en Santé Publique, Birni N’Gaoure, Niger; Centre de Recherche et Interventions en Santé Publique, Birni N’Gaoure, Niger; Centre de Recherche et Interventions en Santé Publique, Birni N’Gaoure, Niger; Centre de Recherche et Interventions en Santé Publique, Birni N’Gaoure, Niger; Francis I. Proctor Foundation, University of California, San Francisco, California, USA; Division of Infectious Diseases & International Health, University of Virginia, Charlottesville, Virginia, USA; School of Public Health, Qingdao University, Qingdao, China; Division of Infectious Diseases & International Health, University of Virginia, Charlottesville, Virginia, USA; Francis I. Proctor Foundation, University of California, San Francisco, California, USA; Department of Ophthalmology, University of California, San Francisco, California, USA; Francis I. Proctor Foundation, University of California, San Francisco, California, USA; Department of Epidemiology and Biostatistics, University of California, San Francisco, California, USA; Department of Ophthalmology, University of California, San Francisco, California, USA; Institute for Global Health Sciences, University of California, San Francisco, California, USA; Francis I. Proctor Foundation, University of California, San Francisco, California, USA; Department of Ophthalmology, University of California, San Francisco, California, USA; Francis I. Proctor Foundation, University of California, San Francisco, California, USA; Department of Epidemiology and Biostatistics, University of California, San Francisco, California, USA; Department of Ophthalmology, University of California, San Francisco, California, USA; Institute for Global Health Sciences, University of California, San Francisco, California, USA; Francis I. Proctor Foundation, University of California, San Francisco, California, USA; Department of Epidemiology and Biostatistics, University of California, San Francisco, California, USA; Department of Ophthalmology, University of California, San Francisco, California, USA; Institute for Global Health Sciences, University of California, San Francisco, California, USA

**Keywords:** spillover, antimicrobial resistance, mass drug administration, azithromycin, Niger

## Abstract

**Background:**

The risk of antibiotic resistance is complicated by the potential for spillover effects from one treated population to another. Azithromycin mass drug administration programs report higher rates of antibiotic resistance among treatment arms in targeted groups. This study aimed to understand the risk of spillover of antibiotic resistance to nontarget groups in these programs.

**Methods:**

Data were used from a cluster-randomized trial comparing the effects of biannual azithromycin and placebo distribution to children 1–59 months old on child mortality rates. Nasopharyngeal samples from untreated children 7–12 years old were tested for genetic determinants of macrolide resistance (primary outcome) and resistance to other antibiotic classes (secondary outcomes). Linear regression was used to compare the community-level mean difference in prevalence by arm at the 24-month time point, adjusting for baseline prevalence.

**Results:**

A total of 1103 children 7–12 years old in 30 communities were included in the analysis (15 azithromycin, 15 placebo). The adjusted mean differences in the prevalence of resistance determinants for macrolides, β-lactams, and tetracyclines were 3.4% (95% confidence interval, −4.1% to 10.8%; *P* = .37), −1.2% (−7.9% to 5.5%; *P* = .72), and −3.3% (−9.5% to 2.8%; *P* = .61), respectively.

**Conclusions:**

We were unable to demonstrate a statistically significant increase in macrolide resistance determinants in untreated groups in an azithromycin mass drug administration program. While the result might be consistent with a small spillover effect, this study was not powered to detect such a small difference. Larger studies are warranted to better quantify the potential for spillover effects within these programs.

## Teaser

A nonsignificant 3.4% increase in community prevalence of macrolide resistance genetic determinants was found in untreated children 7–12 years old within an azithromycin mass drug administration program in Niger. More research is warranted to understand spillover potential within these programs.

## Graphical Abstract

**Graphical Abstract:**
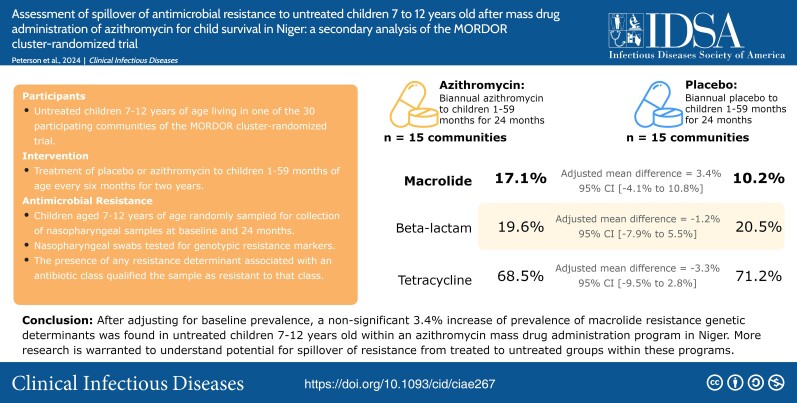
This graphical abstract is also available at Tidbit: https://tidbitapp.io/tidbits/assessment-of-spillover-of-antimicrobial-resistance-to-untreated-children-7-to-12-years-old-after-mass-drug-administration-of-azithromycin-for-child-survival-in-niger-a-secondary-analysis-of-the-mordor-c-d590c4d1-d490-4399-bfba-45e1611c8e80?utm_campaign=tidbitlinkshare&utm_source=ITP

The potential for spillover of antibiotic resistance complicates estimation of the risk of resistance based on antibiotic use [1]. Spillover effects occur through the transmission of antibiotic-resistant bacteria between people, making the risk of antimicrobial resistance (AMR) dependent not only on one's own antibiotic use but also on antibiotic use of the general population [1]. Several observational studies have suggested the presence of spillover effects between family members within a household and among patients in hospitals [2–5]. Ecological and modeling studies have also indicated that spillovers may occur at even larger levels, such as communities or countries, but few studies have explored this phenomenon with empirical data [1, 6–11].

Spillover of antibiotic resistance is a particular concern for mass drug administration (MDA) programs using antibiotics for child survival. MDA programs distributing azithromycin to children 1–59 months old have been found to reduce all-cause child mortality rates but lead to increases in AMR in treated groups [12–15]. MORDOR was a cluster-randomized trial comparing child mortality rates among communities receiving biannual azithromycin or placebo distribution. After 24 months of biannual MDA, the prevalence of macrolide-resistant pneumococcus was higher in the azithromycin arm than in the placebo arm [13]. With continued distributions, at the 36-, 48-, and 60-month time points, macrolide resistance continued to be higher in communities receiving azithromycin compared with placebo [14, 15]. However, studies to date have focused on AMR in groups targeted for treatment with azithromycin. The spillover of resistance from treated to untreated groups within communities receiving azithromycin would indicate that azithromycin MDA may have a larger impact on AMR than previously demonstrated.

More information regarding the effect of spillover within MDA programs is needed to support risk-benefit assessments for this intervention. The placebo-controlled cluster-randomized design of the MORDOR trial is ideal to study spillover [16, 17]. In the current study, we used data collected at the 24-month time point of the MORDOR trial to determine the presence of spillover of genetic determinants of macrolide resistance from eligible children 1–59 months of age to children 7–12 years of age who were not targeted for treatment. We hypothesized that spillover effects would not be identified, given the biannual single-dose administration to a small proportion of the overall population and the 6 months between the last distribution and sample collections.

## METHODS

### Study Design and Data Collection

This study was a prespecified secondary analysis of the MORDOR morbidity trial in the Dosso region of Niger, which has been described elsewhere [13]. In this cluster-randomized trial, 30 communities were randomly selected from the larger pool of MORDOR-eligible communities and randomized to receive biannual azithromycin or placebo distribution. The study team collected census data from each household in the study area and delivered treatment to children 1–59 months old every 6 months for 2 years. Children 7–12 years old in these households were also censused to define the sampling frame for AMR sample collections for this study. Children aged 1–59 months were eligible for treatment if parental consent was received and if they weighed ≥3.8 kg. Treatment dose was determined by weight for children unable to stand and by height for children 12–59 months old who were able to stand. Census data included village, age, and sex. Nasopharyngeal samples were collected from repeated cross-sectional random samples of up to 40 children aged 7–12 years living in these communities, using the most recent census data for sampling at baseline and 24 months. As the aim was to estimate community-level prevalence, the samples obtained at 24 months did not necessarily include the children sampled at baseline. There were no restrictions for how many children in a household could be randomly selected.

Samples were taken by trained examiners using sterile, individually-wrapped pediatric flocked swabs with a plastic swab shaft (Copan Diagnostics). The 24-month collection occurred approximately 6 months after the last MDA. Nasopharyngeal swabs were placed in the child’s right nostril and rotated 180° once they reached the nasopharynx. The swabs were then placed in tubes containing 1.0 mL of DNA/RNA shield (Zymo). Insulated cooler bags with frozen gel ice packs were used to carry samples to and from the field, and samples were then placed in a standard 20°C freezer located at the local health center, which is under 24-hour security guard supervision.

Ethical approval for this study was granted by the Comité Nationale Ethique pour la Recherche en Santé in Niger and the University of California, San Francisco. The study team obtained verbal consent from community and local health center leaders before study activities began, as well as from heads of household and guardians of children in both age groups for census and AMR data collection. Verbal assent was also obtained from children for sample collections. The MORDOR trial was registered at clinicaltrials.gov (NCT02047981).

Nasopharyngeal swab samples were tested for genetic determinants of resistance to macrolides, β-lactams, tetracyclines, and fluoroquinolones using quantitative polymerase chain reaction (PCR) with a customized TaqMan Array Card platform. Briefly, total nucleic acids were extracted with the QIAamp MinElute Virus Spin kit on QIAcube (Qiagen), following the manufacturer's instructions, and eluted in 100 μL. Each of the samples was spiked with external controls (MS2 and PhHV) to monitor the inhibition. One extraction blank was incorporated for up to 36 samples to rule out laboratory contamination. Next, 46 μL of the nucleic acid extract from each sample was mixed with 50 mL of AgPath One-Step reverse-transcription buffer and 4 mL of enzyme mix and then loaded onto each port of a TaqMan Array Card. The PCR analyses were performed on ViiA 7 or QuantStudio 7 real-time PCR systems, with cycling conditions including the reverse-transcription step at 45°C for 20 minutes, initial denaturation at 95°C for 10 minutes, and 40 cycles of 95°C for 15 seconds and 60°C for 1 minute.

A quantification cycle of <30 was considered positive. The results were determined to be invalid and excluded from further analysis when the corresponding external controls or extraction blank failed. The presence of any resistance determinant associated with an antibiotic class qualified the sample as resistant to that class (Supplementary Table 1). Only resistance determinants for which ≥5 individuals had any marker detected were included in analyses because models were unable to converge for markers with <5 data points. If any individual marker was missing data, then the presence of resistance determinants in that class was left missing. The primary outcome was prevalence of genetic determinants of macrolide resistance as indicated by any marker. Secondary outcomes included prevalence of determinants of resistance to β-lactams, fluoroquinolones, and tetracyclines. In addition, the mean quantities of markers summed within antibiotic class and for each individual marker were examined as secondary outcomes. The quantity of a marker was calculated by transforming the cycle threshold values to the copy numbers in log_10_ scale based on the standard curves, with 0 values set to half the limit of detection at the log_10_(0.5) scale [18].

### Statistical Analysis Methods

Sample size was fixed by the primary AMR study examining macrolide resistance in 1–59-month-olds [13]. Inclusion of 15 communities per arm and 40 children per community was anticipated to provide approximately 80% power to detect a 16% difference in prevalence of macrolide resistance determinants, assuming a baseline prevalence of 12%.

Baseline demographic characteristics including age and sex among the children included in sample collections were summarized at the community level and then compared with those for all children aged 7–12 years recorded in the census to determine the representativeness of the sample using permutation *P* values accounting for clustering by community. The primary analysis used a linear regression model to compare community-level mean difference in prevalence of macrolide resistance determinants by arm, adjusting for baseline community prevalence. Similar models were constructed for other antibiotic classes, with community-level adjusted mean difference in prevalence by arm and 95% confidence intervals (CIs) reported for each. A sensitivity analysis was conducted to examine the prevalence of resistance determinants for each class by subgroups of household treatment status, defined by whether a 7–12-year-old was living in a household with a treated 1–59-month-old child. Similar linear regression models were used for this analysis, including terms for the subgroup and interaction between arm and subgroup. Another sensitivity analysis using a higher cutoff of <35 for the quantification cycle was conducted. In addition, paired *t* tests were used to test for differences between baseline and 24-month prevalences for each antibiotic class by arm.

Community-level mean quantities of resistance determinants on the log_10_ scale were compared by arm for each class and individually at the 24-month time point, using linear regression models adjusting for baseline. Resistance determinants where no marker was detected were excluded. As a sensitivity analysis, a permutation test based on the Mann-Whitney *U* statistic was used to compare differences in the quantities for each class and individually by arm. All *P* values were calculated using permutation at the community level with 10 000 permutations. The primary analysis assumed an α value of .05 for statistical significance. Within each set of additional analyses, *P* values were adjusted using Benjamini-Hochberg correction to control the false discovery rate at 5% [19].

## RESULTS

**Figure 1.**
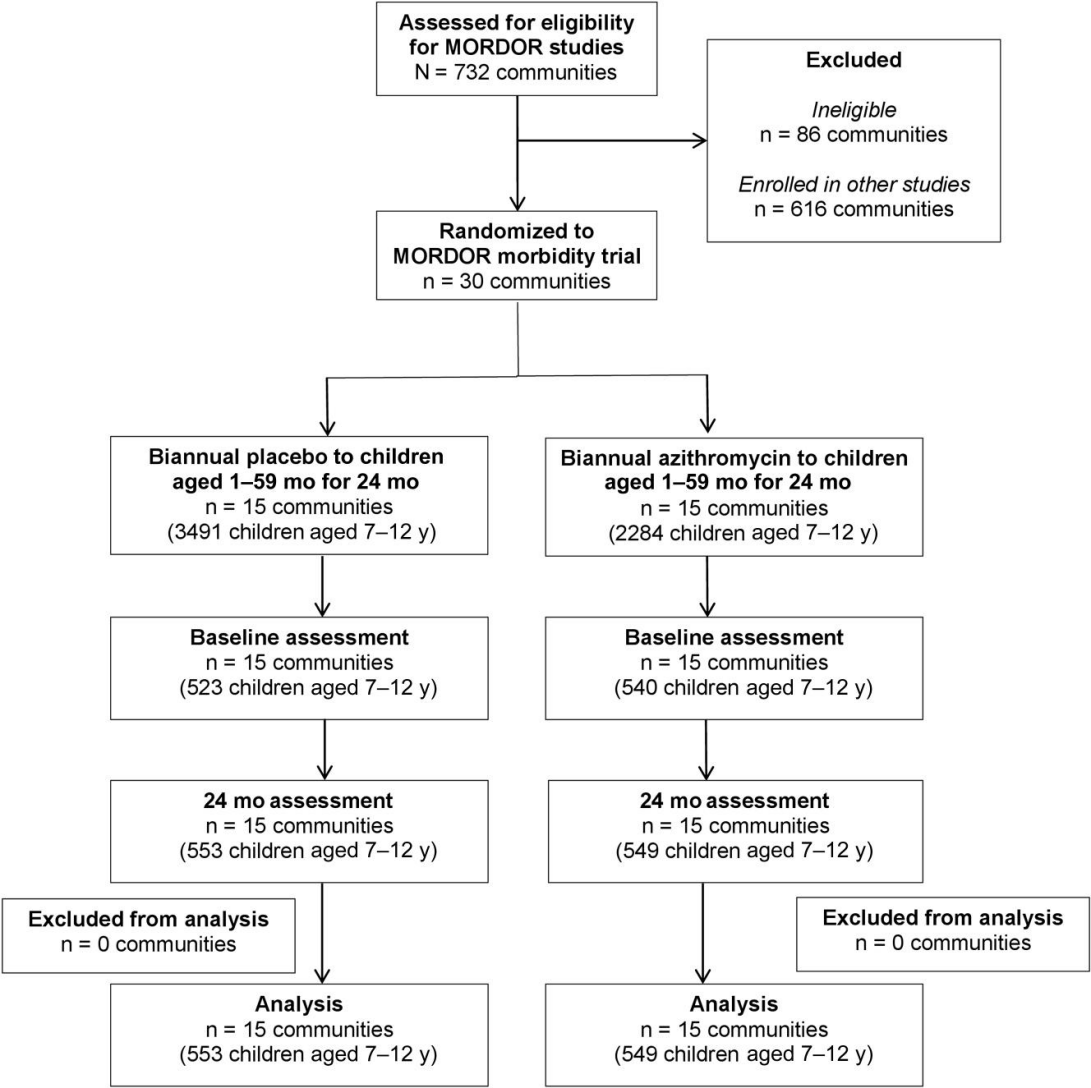
Participant flow for the current study.

Baseline sample collection occurred from March to June 2015, and 24-month sample collection from March to June 2017 in 30 communities. Samples were obtained from 1066 children at baseline and 1103 at 24 months (Figure 1). Overall, baseline characteristics were similar by arm (Table 1). At baseline, 50.7% of children were female, and the mean age (standard deviation) was 8.8 (0.3) years. The prevalence of macrolide resistance determinants at baseline was 9.1% (95% CI, 5.1%–13.1%) in the azithromycin arm and 5.0% (2.7%–7.2%) in the placebo arm (Table 1). At baseline, 3 children were missing data for β-lactam resistance markers and 1 for fluoroquinolone resistance markers, all in the placebo arm. At 24 months, 1 child was missing data for β-lactam resistance markers in the placebo arm. The community-level age and sex of children included in sample collections were similar to all children 7–12 years old included in the census (Supplementary Table 2).

**Table 1.** Community-Level Characteristics of Untreated Children 7–12 Years Old in the MORDOR Morbidity Trial in Niger at Baseline

Characteristic	Azithromycin Arm (n = 15 Communities)	Placebo Arm (n = 15 Communities)	Overall (n = 30 Communities)
Untreated children aged 7–12 y, no.	549	553	1102
Age, mean (SD)	8.9 (0.2)	8.8 (0.3)	8.8 (0.3)
Female sex, mean (SD), %	47.7 (11.2)	53.8 (6.6)	50.7 (9.6)
Prevalence of resistance, mean (SD), %			
Macrolides	9.1 (7.2)	5.0 (4.1)	7.0 (6.1)
β-Lactams	10.4 (5.7)	13.0 (7.7)	11.7 (6.7)
Tetracyclines	63.2 (10.1)	60.5 (15.7)	61.8 (13.0)
Fluoroquinolones	0 (0)	0 (0)	0 (0)

Abbreviation: SD, standard deviation.

**Figure 2.**
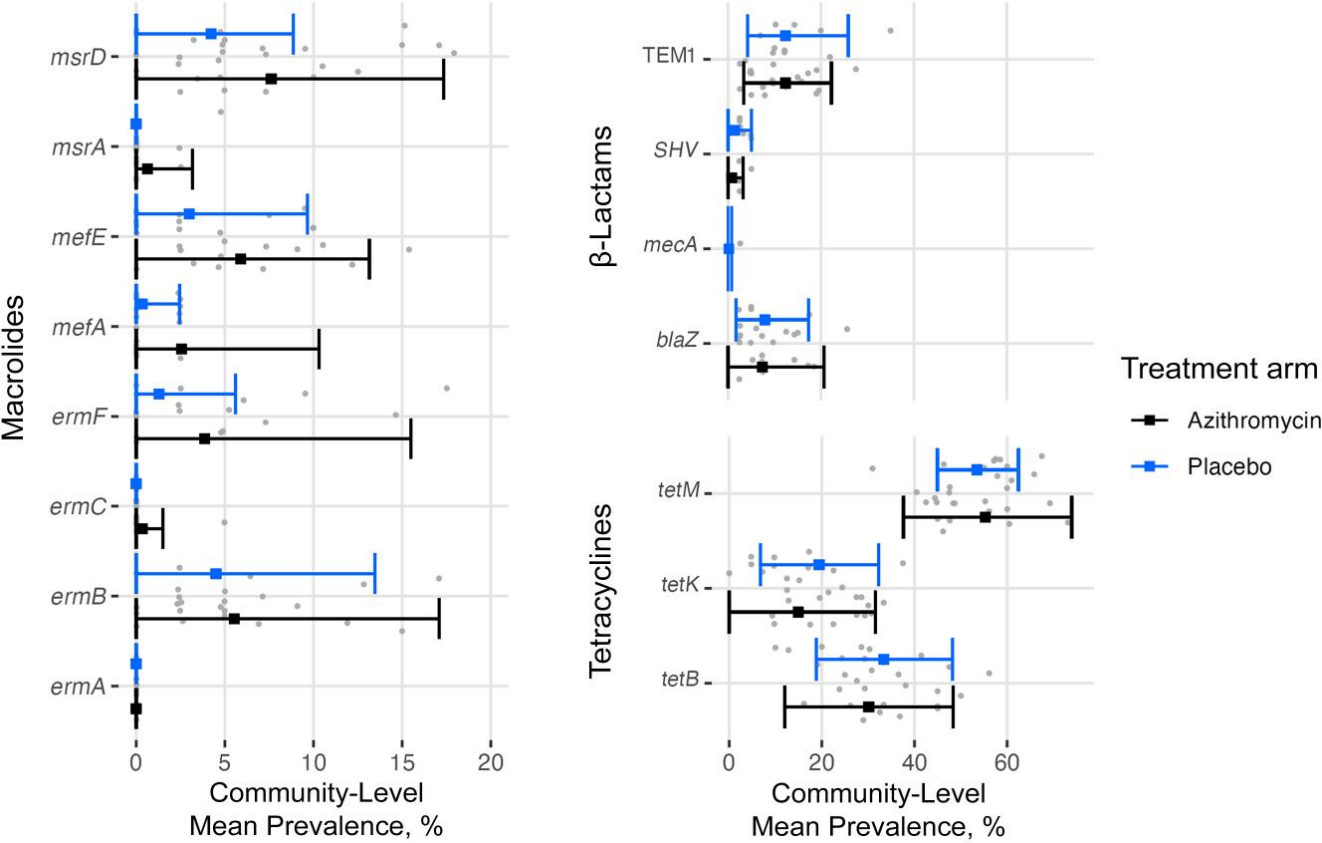
Prevalence of individual resistance markers by antibiotic class and treatment arm among children 7–12 years old at 24 months in the MORDOR morbidity trial in Niger. Gray dots represent individual community prevalence. The x-axis for the macrolide class differs from those for the β-lactam and tetracycline classes. Fluoroquinolones are not shown, as no quantities were detected in either arm.

The prevalence of macrolide resistance determinants at 24 months among children 7–12 years old was 17.1% (95% CI, 9.8%–24.4%) in the azithromycin and 10.2% (5.8%–14.6%) in the placebo arm (adjusted mean difference, 3.4% [−4.1% to 10.8%]; *P* = .37) (Table 2). The adjusted mean differences in prevalence of resistance determinants for β-lactams and tetracyclines were −1.2% (95% CI, −7.9% to 5.5%; *P* = .72) and −3.3% (−9.5% to 2.8%; *P* = .61), respectively. No prevalence of resistance determinants for fluoroquinolones was detected in either arm. We found no evidence of a differential effect of treatment arm on prevalence by presence of a treated child in the household (Supplementary Table 3). The proportion of children living in a treated household was 60.7% for the azithromycin arm, 66.9% for the placebo arm, and 63.8% overall. Using a higher cutoff of <35 for the quantification cycle did not alter the interpretation of mean differences (Supplementary Table 4). The prevalence of individual resistance markers by antibiotic class is shown in Figure 2. The prevalence of resistant determinants of macrolide class increased by 8.0% (95% CI, 1.9%–14.1%; *P* = .01) in the azithromycin arm and by 5.25% (.8%–9.8%; *P* = .02) in the placebo arm between baseline and the 24-month time point (Supplementary Table 5).

**Table 2.** Difference in Community-Level Mean Prevalence of Genetic Determinants of Resistance to 4 Antibiotic Classes at 24 Months in Untreated Children 7–12 Years Old in the MORDOR Morbidity Trial in Niger

Antibiotic Class	Mean Prevalence (SD)		Adjusted Mean Difference (95% CI), %^a^	*P* Value
	Azithromycin (n = 15 Communities)	Placebo (n = 15 Communities)		
Macrolides	17.1 (13.2)	10.2 (8.0)	3.4 (4.1–10.8)	.37
β-Lactams	19.6 (7.8)	20.5 (10.3)	−1.2 (−7.9 to 5.5)	.72
Fluoroquinolones	0 (0)	0 (0)	0 (0–0)	…
Tetracyclines	68.5 (9.7)	71.2 (8.2)	−3.3 (−9.5 to 2.8)	.61

Abbreviations: CI, confidence interval; SD, standard deviation.

^a^Estimated with community-level linear regression with adjustment for baseline community prevalence.

**Figure 3.**
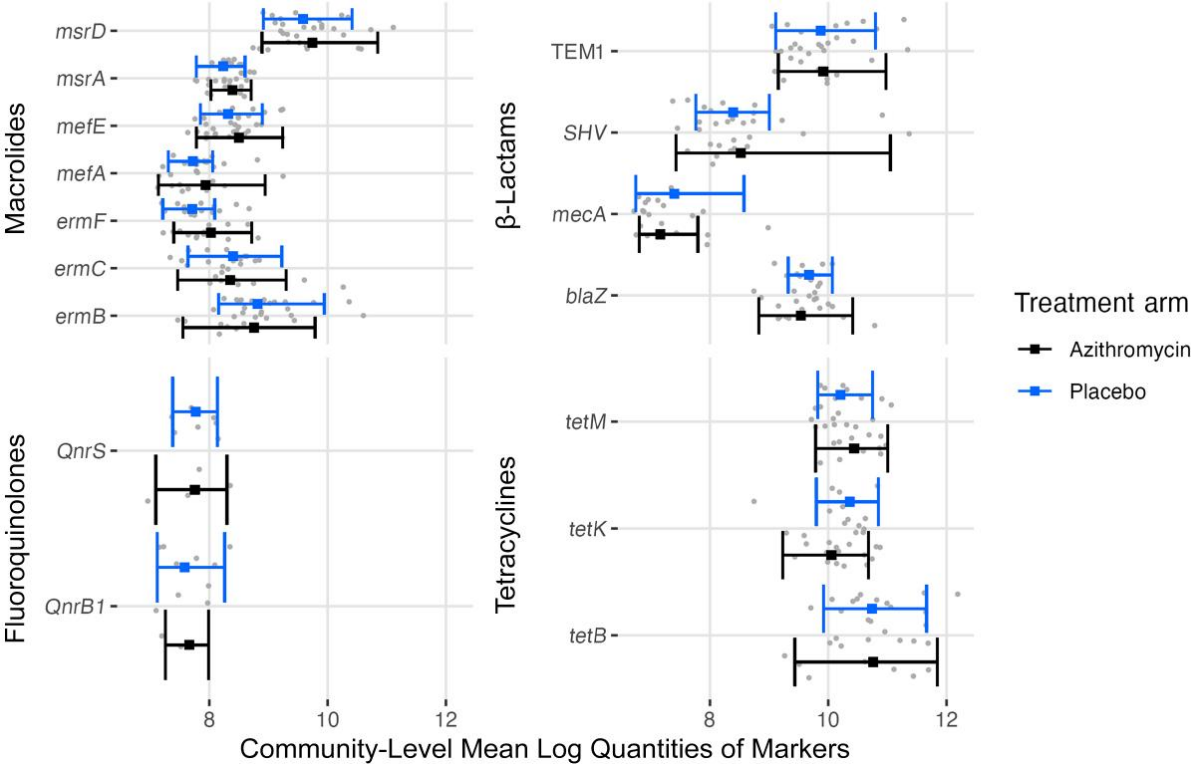
Quantities of individual resistance markers by antibiotic class and treatment arm among children 7–12 years old at 24 months in the MORDOR morbidity trial in Niger.

No notable differences were identified when comparing the sum quantities of genetic resistance determinants detected for each antibiotic class by arm at the 24-month time point (Table 3). Sensitivity analyses found similar results (Supplementary Table 6). Figure 3 displays the quantity for each individual marker in each of the 4 classes by arm. When compared by arm at the 24-month time point, none of the resistance markers had significantly different quantities (Table 4). Another sensitivity set of analyses similarly found no differences when comparing quantities of individual markers detected by arm (Supplementary Table 7).

**Table 3.** Adjusted Mean Difference in Log Quantities of Resistance Markers by Treatment Arm for 4 Antibiotic Classes at 24 Months in Children 7–12 Years Old in the MORDOR Morbidity Trial in Niger

Antibiotic Class	Log Quantity of Resistance Markers, Mean (SD)		Adjusted Mean Difference in Quantities (95% CI)^a^	*P* Value
	Azithromycin	Placebo		
Macrolides	127.27 (38.46)	114.54 (29.38)	5.72 (−14.18 to 25.62)	.78
β-Lactams	130.89 (28.98)	130.32 (36.29)	0.15 (−22.48 to 22.78)	.99
Fluoroquinolones	2.23 (3.21)	4.93 (4.08)	−2.76 (−5.51 to −.02)	.19
Tetracyclines	181.49 (41.30)	185.39 (44.32)	−8.36 (−33.53 to 16.82)	.78

Abbreviations: CI, confidence interval; SD, standard deviation.

^a^Estimated with community-level linear regression with adjustment for baseline community mean quantity.

**Table 4.** Adjusted Mean Difference in Individual Log Quantities by Resistance Marker and Treatment Arm at 24 Months in Children 7–12 Years Old in the MORDOR Morbidity Trial in Niger Adjusted for Baseline Quantity

Resistance Markers by Antibiotic Class^a^	Log Quantity of Resistance Markers, Mean (SD)		Adjusted Mean Difference in Quantity (95% CI)	*P* Value
	Azithromycin Arm	Placebo Arm		
Macrolides				
*ermB*	8.76 (.75)	8.82 (.65)	−0.08 (−.62 to .46)	.90
*ermC*	8.35 (.68)	8.4 (.58)	0.07 (−.55 to .68)	.90
*ermF*	8.03 (.49)	7.71 (.29)	0.27 (.03–.52)	.48
*mefA*	7.93 (.57)	7.72 (.26)	0.05 (−.24 to .34)	.90
*mefE*	8.5 (.49)	8.32 (.39)	0.16 (−.16 to .48)	.90
*msrA*	8.39 (.23)	8.24 (.27)	0.15 (−.04 to .33)	.64
*msrD*	9.74 (.68)	9.58 (.53)	0.17 (−.27 to .62)	.90
β-Lactams				
*blaZ*	9.54 (.56)	9.67 (.24)	−0.13 (−.45 to .18)	.90
*mecA*	7.16 (.36)	7.4 (.77)	−0.05 (−.69 to .60)	.90
SHV	8.52 (1.17)	8.39 (.46)	0.15 (−.45 to .75)	.90
TEM1	9.92 (.66)	9.87 (.6)	0.14 (−.33 to .61)	.90
Tetracyclines				
*tetB*	10.76 (.83)	10.74 (.67)	0.06 (−.48 to .60)	.90
*tetK*	10.05 (.53)	10.36 (.41)	0−.28 (−.61 to .06)	.64
*tetM*	10.44 (.47)	10.21 (.31)	0.19 (−.11 to .50)	.72

Abbreviations: CI, confidence interval; SD, standard deviation.

^a^The following markers had low or no quantities detected: *ermA, CTX-M1, CTX_M9, CTX_M8_M25, CTX_M2_M74, QnrA, QnrB1, QnrS,* and *QnrB4*.

## DISCUSSION

In this secondary analysis of a cluster-randomized trial, we compared the prevalence of genetic determinants of macrolide resistance among untreated children in communities randomized to receive biannual azithromycin or placebo MDA over 2 years. We were unable to demonstrate a difference in macrolide resistance prevalence by arm, although the study was not powered to detect a 3.4% increase. While this result is potentially consistent with a small spillover, a larger study would be needed to demonstrate an effect of this size. Additional analyses showed no increases in quantities of individual resistance determinants in azithromycin communities compared with placebo communities.

Although we were unable to detect a difference in macrolide resistance prevalence by arm, an increase in resistance in untreated groups in the same community is plausible. Previous work on antibiotic resistance spillovers has highlighted the role of population interaction in influencing spillover effects among populations with different levels of antibiotic use. Simulations demonstrated that greater interaction among 2 populations increased resistance within the group with lower antibiotic use more than if these populations had interacted less frequently [1]. In this study, the selected untreated group was not required to be living in a household with a treated child, allowing us to examine a community-level effect. Thus, interaction among the treated and untreated children may have been limited, especially since the treated group was not yet old enough to attend school. Still, 64% of 7–12-year-olds selected at the 24-month time point lived in a household with ≥1 child aged 1–59 months who was treated at any time in the study, suggesting frequent household level interaction. In addition, each community was treated every 6 months, and 24-month sample collection for this study occurred 6 months after the most recent distribution. It is possible that any spillover effect had waned in the time since treatment.

A systematic review of trachoma control programs using azithromycin MDA found evidence that the prevalence of resistance decreases after termination of the antibiotic use [20]. Overall, while there may be potential for spillover of resistance, the World Health Organization has determined that the actual morbidity and mortality risk of the AMR found after MDA of azithromycin remains unclear and that the mortality benefit of these MDA programs outweighs the AMR risk given this uncertainty [21]. If these programs do produce a small spillover effect to the untreated groups, questions regarding the clinical impact and duration of this effect will remain.

While the macrolide class was of primary interest, nonmacrolide antibiotic classes were also included in the analysis. β-Lactam resistance is especially of interest, as this class of antibiotics is widely used in this setting and waning efficacy would have significant consequences [22]. Results of the MORDOR trial suggested that β-lactam resistance may have increased in azithromycin-treated communities compared with placebo-treated communities at 36 months, but no increase was shown at the 5-year time point [14, 15]. In the present study we found no evidence of an increase in nonmacrolide resistance determinants in untreated children after 24 months of MDA. We did find evidence of an increase in resistance to most antibiotic classes from baseline to the 24-month time point in the untreated population. Although we were unable to examine this further here, this increase may be due to the increasing use of antibiotics in the study area in general over time. There is also a possibility of resistance transmission between communities, considering how azithromycin and placebo communities were interspersed, but since we found no overall difference between arms this seems less likely.

Strengths of the current study include the randomized controlled trial design and outcome prespecification, which diminish the potential for bias. The use of the TaqMan Array Card allowed for efficient screening of resistance determinants among all 4 antibiotic classes, and genotypic approaches have been recommended for surveillance of antibiotic resistance after azithromycin MDA [23]. There was little missing data overall. The sample collections also occurred in the same time period for baseline and at 24 months, limiting the influence of any differences based on seasonality.

One limitation of this study is the choice of macrolide genes tested and their clinical relevance. First, use of a targeted PCR approach limited the number of assays we could include on the TaqMan Array Card platform. We therefore chose the most common macrolide resistance genes that are known to confer resistance to clinically relevant pathogens that reside in the nasopharynx, such as *Streptococcus pneumoniae* (*ermB, mefA, mefE,* and *msrD*), *Hemophilus influenzae* (additionally *ermF*), *Staphylococcus aureus* (additionally *ermC* and *msrA*), and *Streptococcus pyogenes* (*ermF* and *msrD*) [24–29]. One can debate the choice of gene targets and the clinical significance of such carriage. We believe, however, that this assessment of 7 of the most important macrolide resistance determinants was a reasonable approach to examine the potential for clinically significant macrolide resistance to develop after MDA. Of course, the use of a genotypic approach precludes the ability to identify the specific organisms carrying resistance.

Further limitations include the fact that the untreated population was not necessarily living in households with treated children. Although this facilitates assessing a larger community-level effect, it is also possible that these older children mainly interact with younger children if they share a household. In addition, adults in the household responsible for caring for children 1–59 months old are likely to have the most interaction with the treated population. Although we did not have the resources to include adults in this study, other studies currently underway are including adult groups in sample collections [30]. Similarly, although this study did not include a focus on resistance determinants in gastrointestinal pathogens in a nontarget group, this is being explored elsewhere [30]. It is also possible that antibiotic resistance in untreated groups increases over time, so longer term follow-up would be needed. Finally, this study was not powered to detect small differences by arm.

Overall, we were unable to demonstrate spillover of macrolide resistance determinants to untreated children in communities receiving biannual azithromycin MDA compared with placebo. As this study was underpowered to detect small effects, we cannot definitely rule out the possibility of small spillover effects of azithromycin MDA to untreated groups. Future larger studies powered to detect smaller spillover effects are warranted.

## Supplementary Data

Supplementary materials are available at *Clinical Infectious Diseases* online. Consisting of data provided by the authors to benefit the reader, the posted materials are not copyedited and are the sole responsibility of the authors, so questions or comments should be addressed to the corresponding author.

## Supplementary Material

ciae267_Supplementary_Data

## Notes

***Author Contributions.*** A. M. A., A. A., R. M., T. D., T. C. P., J. D. K., T. M. L., and K. S. O. contributed to the study design. A. M. A., R. M., A. M. K., M. M., M. K., Z. L., E. H., J. L., S. P., T. D., J. D. K., and T. M. L. contributed to study implementation, data collection, and/or specimen processing. B. P. contributed to data analyses. B. P. and K. S. O. drafted the manuscript, and all authors approved the final version of the manuscript.

***Financial support***. This work was supported by the Bill & Melinda Gates Foundation (grant OP1032340) and the National Institute of Allergy and Infectious Disease, National Institutes of Health (grant R01AI175250).
