# Supplementary material for: Assessment of Spillover of Antimicrobial Resistance to Untreated Children 7–12 Years Old After Mass Drug Administration of Azithromycin for Child Survival in Niger: A Secondary Analysis of the MORDOR Cluster-Randomized Trial
Source: Clin Infect Dis. 2024 May 13;79(5):1136–43. doi: 10.1093/cid/ciae267 (PMC11581702; doi:10.1093/cid/ciae267)
Supplement: ciae267_Supplementary_Data [file ciae267_supplementary_data.docx]

**Supplemental Tables and Figures**

Supplemental Table 1. Markers of genetic determinants of resistance by class.

| **Antibiotic Class** | **Genetic Determinant of Resistance Marker** | **Pathogenic bacteria which commonly harbor these genes** |
| --- | --- | --- |
| **Macrolide** |  |  |
|  | ermA | *S. aureus* |
|  | ermB | *S. pneumoniae, H. influenzae, S. pyogenes* |
|  | ermC | *S. aureus* |
|  | ermF | *H. influenzae* |
|  | mefA | *S. pneumoniae, H. influenzae, S. pyogenes* |
|  | mefE | *S. pneumoniae* |
|  | msrA | *S. aureus* |
|  | msrD | *S. pyogenes* |
| **Beta-Lactam** |  |  |
|  | blaZ | *S. aureus* |
|  | CTX-M | *K.pneumoniae, E.coli* |
|  | CTX_M2_M74 | *K.pneumoniae* |
|  | CTX_M8_M25 | *E.coli* |
|  | CTX_M9 | *K.pneumoniae, E.coli* |
|  | mecA | *S. aureus* |
|  | SHV | *K.pneumoniae, E.coli* |
|  | TEM_1 | *H. influenzae, K. pneumoniae, E. coli* |
| **Fluoroquinolone** |  |  |
|  | QnrA | *K. pneumoniae, E. coli* |
|  | QnrS | *K. pneumoniae, E. coli* |
|  | QnrB1 | *K. pneumoniae, E. coli* |
|  | QnrB4 | *K. pneumoniae, E. coli* |
| **Tetracycline** |  |  |
|  | tetB | *H. influenzae, S. aureus, S. pyogenes, M. catarrhalis, M. pneumoniae* |
|  | tetK | *S. aureus* |
|  | tetM | *S. pneumoniae* |

Supplemental Table 2. Comparison of community-level baseline characteristics among children 7-12 years old included in sample collections and among those censused overall

| **Characteristic** | **Included in Sample Collections** | | | **Overall Census Participants** | | | **P value** |
| --- | --- | --- | --- | --- | --- | --- | --- |
|  | Azithromycin  n = 542 | Placebo  n = 524 | Overall  n = 1,066 | Azithromycin  n = 44,195 | Placebo  n = 72,598 | Overall  n = 116,793 |  |
| Communities  n (%) | 15 (50%) | 15 (50%) | 30 (100%) | 15 (50%) | 15 (50%) | 30 (100%) |  |
| Age  Mean (SD) | 8.9 (0.3) | 8.8 (0.4) | 8.8 (0.3) | 9.0 (0.3) | 9.0 (0.2) | 9.0 (0.3) | 0.051 |
| Percent female  n (%) | 47.7% (11.2%) | 53.8% (6.6%) | 50.7% (9.6%) | 46.2% (7.6%) | 47.6% (4.3%) | 46.9% (6.1%) | 0.07 |

Supplemental Table 3. Difference in mean prevalence of genetic determinants of resistance to each antibiotic class at 24 months by household treatment status adjusting for baseline prevalence.

|  | **Treated Households**  703 (63.8%) | | **Untreated Households**  399 (36.2%) | |  |
| --- | --- | --- | --- | --- | --- |
| **Resistance Class** | **Mean Difference in Prevalence** | **95% Confidence Interval** | **Mean Difference**  **In Prevalence** | **95% Confidence Interval** | **P-value for interaction** |
| Macrolide | 2.6% | -6.0% to 11.2% | 3.4% | -4.9% to 11.8% | 0.81 |
| Beta-lactam | -0.1% | -6.9% to 6.7% | -6.4% | -17.1% to 4.3% | 0.39 |
| Fluoroquinolone | 0% | 0% to 0% | 0% | 0% to 0% | - |
| Tetracycline | -0.5% | -6.8% to 5.8% | -8.2% | -18.6% to 2.0% | 0.39 |

Supplemental Table 4. Difference in community-level mean prevalence of genetic determinants of resistance to four antibiotic classes at 24 months among untreated children 7-12 years old in the MORDOR Morbidity trial in Niger using a higher cutoff for the quantification cycle of < 35.

|  | **Azithromycin**  **Mean prevalence (SD)**  **N = 15 communities** | **Placebo**  **Mean prevalence (SD)**  **N = 15 communities** | **Adjusted Mean Difference*** | **95% Confidence Interval** | **P-value** |
| --- | --- | --- | --- | --- | --- |
| **Macrolide** | 82.7% (13.3%) | 78.5% (6.7%) | 2.7% | -3.8% to 9.3% | 0.43 |
| **Beta-lactam** | 80.5% (9.0%) | 78.8% (9.2%) | 1.6% | -4.9% to 8.1% | 0.83 |
| **Fluoroquinolone** | 1.8% (2.5%) | 3.8% (3.1%) | -2.1% | -4.2% to -0.03% | 0.18 |
| **Tetracycline** | 96.9% (3.7%) | 97.1% (3.0%) | -0.3% | -2.7% to 2.2% | 0.83 |

* Estimated with community-level linear regression adjusting for baseline community prevalence.

Supplemental Table 5. Comparison of the community-level prevalence of resistance determinants to each antibiotic class at baseline and 24-month timepoint within each arm.

| **Class** | **Azithromycin** | | **Placebo** | |
| --- | --- | --- | --- | --- |
|  | **Mean Difference (95% CI)** | **P-value** | **Mean Difference (95% CI)** | **P-value** |
| Macrolide | 8.0%  (1.9% to 14.1%) | 0.010 | 5.3%  (0.8% to 9.8%) | 0.018 |
| Beta-lactam | 9.2%  (4.0% to 14.4%) | 0.002 | 7.5%  (-0.3% to 15.2%) | 0.062 |
| Fluoroquinolone | 0% (0% to 0%) | - | 0% (0% to 0%) | - |
| Tetracycline | 5.3%  (-2.2% to 12.8%) | 0.156 | 10.7%  (3.7% to 17.8%) | 0.005 |

Supplemental Table 6. Sensitivity analysis of the difference in quantities of resistance markers by class using Mann Whitney U Statistic

|  | **Azithromycin**  **Median (IQR)** | **Placebo**  **Median (IQR)** | **P value** |
| --- | --- | --- | --- |
| **Macrolide** | 4.12 (3.65, 4.61) | 3.88 (3.45, 4.36) | 0.44 |
| **Beta-lactam** | 4.24 (3.9, 4.77) | 4.21 (3.84, 4.83) | 0.79 |
| **Fluoroquinolone** | 3.38 (3.23, 3.47) | 3.39 (3.17, 3.52) | 0.22 |
| **Tetracycline** | 5.04 (4.49, 5.7) | 5.12 (4.59, 5.71) | 0.79 |

Supplemental Table 7. Sensitivity analysis of the difference in quantities of individual resistance markers using Mann Whitney U Statistic

| **Resistance Class** | **Resistance Marker** | **Azithromycin**  **Median (IQR)** | **Placebo Median (IQR)** | **P value** |
| --- | --- | --- | --- | --- |
| **Macrolide** |  |  |  |  |
|  | ermB | 8.78 (8.46, 9) | 8.59 (8.38, 9.19) | 0.95 |
|  | ermC | 8.26 (8.13, 8.55) | 8.49 (7.99, 8.64) | 0.85 |
|  | ermF | 7.87 (7.67, 8.39) | 7.76 (7.54, 7.91) | 0.51 |
|  | mefA | 7.92 (7.64, 8.15) | 7.78 (7.54, 7.93) | 0.62 |
|  | mefE | 8.49 (8.21, 8.7) | 8.34 (8.03, 8.57) | 0.62 |
|  | msrA | 8.38 (8.26, 8.54) | 8.27 (8.01, 8.44) | 0.51 |
|  | msrD | 9.64 (9.22, 10.17) | 9.48 (9.22, 9.88) | 0.85 |
| **Beta-lactam** |  |  |  |  |
|  | blaZ | 9.47 (9.16, 9.88) | 9.7 (9.49, 9.81) | 0.62 |
|  | mecA | 7.12 (6.9, 7.28) | 6.97 (6.83, 7.95) | 0.93 |
|  | SHV | 8.11 (7.86, 8.7) | 8.4 (8.12, 8.6) | 0.82 |
|  | TEM_1 | 9.93 (9.37, 10.14) | 9.83 (9.45, 10.22) | 0.95 |
| **Tetracycline** |  |  |  |  |
|  | tetB | 10.72  (10.45, 11.33) | 10.68  (10.18, 11.25) | 0.95 |
|  | tetK | 10.07  (9.8, 10.4) | 10.36  (10.16, 10.62) | 0.51 |
|  | tetM | 10.56  (10.02, 10.88) | 10.17  (10.02, 10.36) | 0.62 |

These markers had low or no quantities detected: ermA, CTX-M, CTX_M9, CTX_M8_M25, CTX_M2_M74, QnrA, QnrB1, QnrS, and QnrB4.
